# B-periodic oscillations in the Hall-resistance induced by a dc-current-bias under combined microwave-excitation and dc-current bias in the GaAs/AlGaAs 2D system

**DOI:** 10.1038/s41598-018-26009-z

**Published:** 2018-05-18

**Authors:** Han-Chun Liu, C. Reichl, W. Wegscheider, R. G. Mani

**Affiliations:** 1 Department of Physics and Astronomy, Georgia State University, Atlanta, 30303 Georgia; 2 Laboratorium für Festkörperphysik, ETH Zürich, Zürich, CH-8093 Switzerland

## Abstract

We report the observation of dc-current-bias-induced *B*-periodic Hall resistance oscillations and Hall plateaus in the GaAs/AlGaAs 2D system under combined microwave radiation- and dc bias excitation at liquid helium temperatures. The Hall resistance oscillations and plateaus appear together with concomitant oscillations also in the diagonal magnetoresistance. The periods of Hall and diagonal resistance oscillations are nearly identical, and source power (*P*) dependent measurements demonstrate sub-linear relationship of the oscillation amplitude with *P* over the span 0 < P ≤ 20 mW.

## Introduction

Magnetotransport studies of two-dimensional electron systems (2DES) subjected to microwave, mm-wave, and terahertz photoexcitation have revealed many interesting phenomena including the radiation-induced zero-resistance states and associated radiation-induced magnetoresistance oscillations, which have drawn attention from both experiment and theory^1–48^. It is by now well-known that the above mentioned radiation-induced magnetotransport effect consists of 1/4-cycle phase-shifted 1/*B*-periodic oscillations, where the oscillatory minima emerge in the vicinity of *B* = [4/(4*j* + 1)]*B*_*f*_, where *B*_*f*_ = 2πfm^*^/*e*, *f* is the microwave frequency, m^*^ is the effective electron mass and *j* = 1, 2, 3…. Such oscillatory magnetoresistance is mostly observed, at modest radiation-intensity, in the regime of approximately 2*πf* > *ω*_*c*_, where *ω*_*c*_ is the cyclotron frequency. It turns out that, in addition to the above mentioned 1/*B* periodic photo-excited magnetotransport effects, there are also *B*-periodic oscillatory photo-excited magneto-oscillations in both the diagonal resistance, *R*_*xx*_, and the photo-voltage *V*_*p*_. In contrast to 1/*B* periodic photo-excited magnetotransport effects which occur approximately when 2*πf* > *ω*_*c*_, these *B*-periodic magneto-oscillations in the *R*_*xx*_ and *V*_*p*_ are typically observed at 2*πf* < *ω*_*c*_, i.e., *B* > *B*_*f*_^49–52^ Further, initial reports^49,50^ proposed that the oscillation period, Δ*B*, follows Δ*B*∝*n*_*e*_/*ωL*, where *n*_*e*_ is electron density and *L* is the distance between potential probes along the Hall bar. Such oscillations in *R*_*xx*_ and *V*_*p*_ were attributed to the interference of coherently excited edge magnetoplasmons (EMP) at contacts along the periphery of the sample^49,50,53,54^. In their study, Stone *et al*.^52^ confirmed the existence of such *B*-periodic oscillations in the regime 2*πf* < *ω*_*c*_, in specimens where both the 1/*B* periodic and the *B* periodic photo-excited magneto oscillations occur together. However, they found that the period Δ*B* is independent of *L*, the spacing between adjacent contacts^52^, which suggested a reduced role for the interference between edge magnetoplasmons excited at adjacent contacts, and generally pointed to effects within a contact.

Here, we report the observation of *B*-periodic oscillations, Δ*R*_*xy*_, in the Hall resistance, *R*_*xy*_, which go together, remarkably, with plateau-like features in the Hall resistance trace, and examine the correlation of these Δ*R*_*xy*_ oscillations with *B*-periodic diagonal magnetoresistance oscillations, Δ*R*_*xx*_, induced by microwave photo-excitation. Critically, it turns out that the realization of such *B*-periodic oscillations in both *R*_*xy*_ and *R*_*xx*_ in our specimens requires the injection of a supplemental dc-current, *I*_*dc*_, into the specimen. The observed *B*-periodic oscillations in Δ*R*_*xy*_ and Δ*R*_*xx*_ appear very similar, although the Δ*R*_*xy*_ oscillations are larger in magnitude, their amplitudes increase sub-linearly with the microwave power, and the period Δ*B* decreases with increasing microwave frequency, *f*. The necessity of a supplemental *dc*-current for the observability of this effect suggests a role for heating in this observed 2DES effect.

## Results

**Figure 1:**
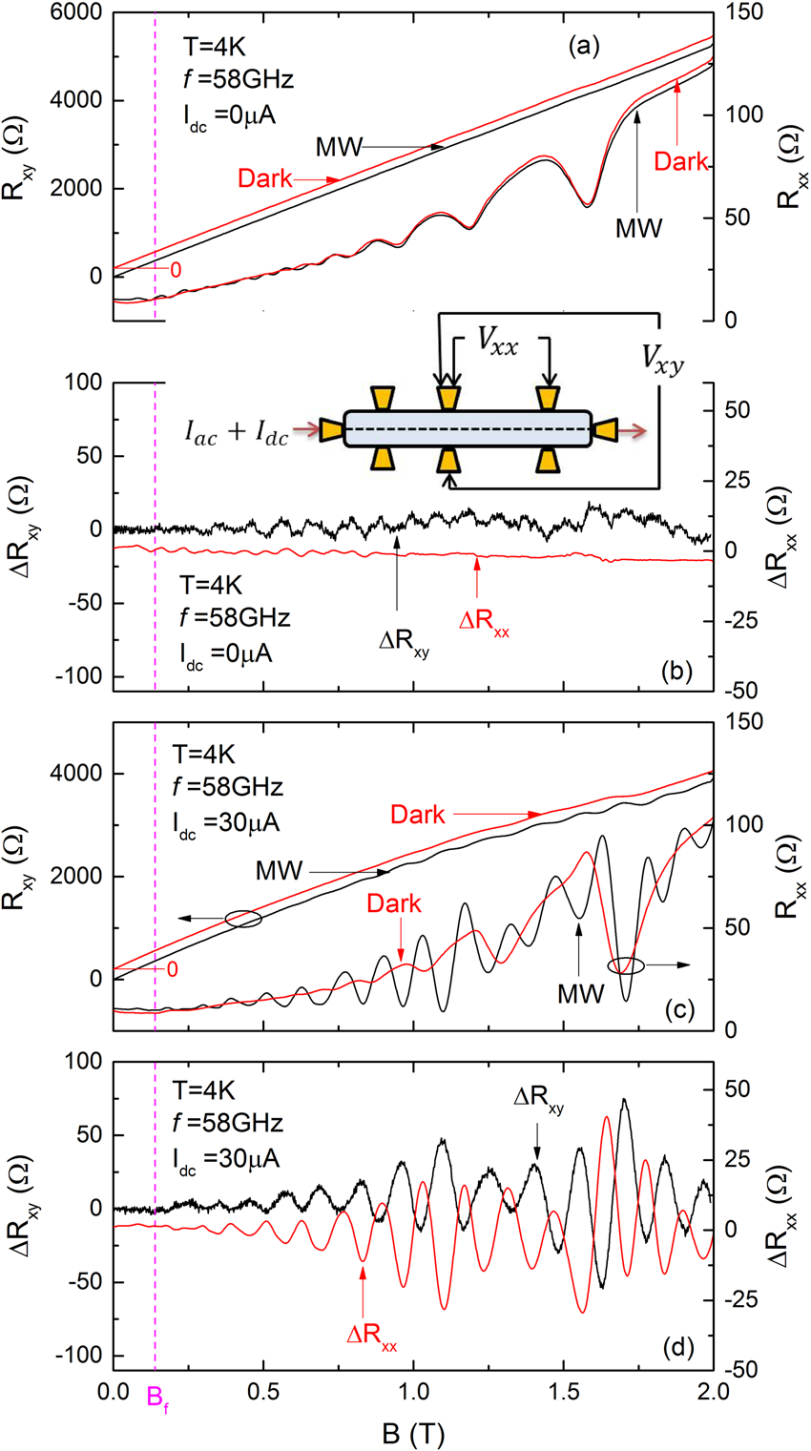
The inset shows a schematic of the sample and the measurement configuration. (**a**) The Hall resistance, *R*_*xy*_, and diagonal resistance, *R*_*xx*_, measured under both dark- and photo-excited (*f* = 58 *GHz*) conditions with *I*_*dc*_ = 0 *μA*. The dark *R*_*xy*_ has been offset with respect to the photo-excited curve for the sake of clarity. The characteristic field of cyclotron resonance, labeled as *B*_*f*_, is indicated by the dashed vertical line. (**b**) This panel shows the difference between the photo-excited and dark resistances shown in Fig. 1(a), i.e., ${\rm{\Delta }}{R}_{xy}={R}_{xy}^{(photo-excited)}-{R}_{xy}^{(dark)}$, and ${\rm{\Delta }}{R}_{xx}={R}_{xx}^{(photo-excited)}-{R}_{xx}^{(dark)}$. (**c**) The Hall resistance, *R*_*xy*_, and diagonal resistance, *R*_*xx*_, measured under both dark- and photo-excited (*f* = 58 *GHz*) conditions with a supplemental *I*_*dc*_ = 30 *μA*. Upon applying *I*_*dc*_ = 30 *μA*, the photo-excited *R*_*xy*_ starts exhibiting *B*-periodic oscillations at high *B*. Concurrently, *B*-periodic oscillations appear on the SdH oscillations in *R*_*xx*_. (**d**) Δ*R*_*xy*_ (left ordinate) and Δ*R*_*xx*_ (right ordinate) obtained from the data of Fig. 1(c) are plotted vs. B. The results suggest anti-phase B-perioidic oscillations in Δ*R*_*xy*_ and Δ*R*_*xx*_.

**Figure 2:**
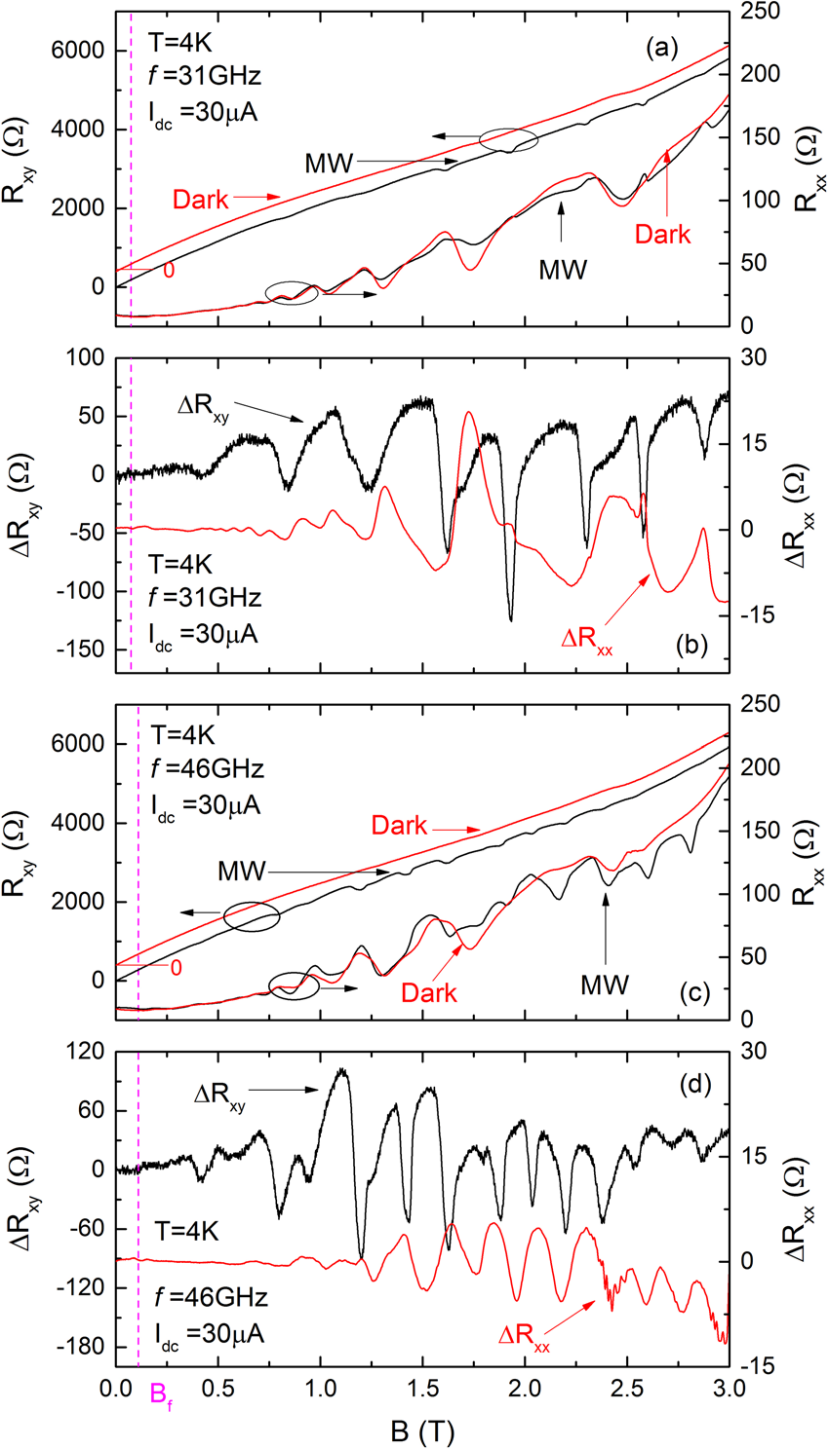
The photo-excited- and dark- curves of *R*_*xy*_ and *R*_*xx*_ at *I*_*dc*_ = 30 *μA*, and (**a**) *f* = 31 and (**c**) *f* = 46 *GHz*. Panel (b) and (d) show Δ*R*_*xy*_ and Δ*R*_*xx*_ at *f* = 31 and 46 *GHz* respectively. Note the variation of the period of the *B*-periodic oscillations with *f*.

Figure 1(a) shows the dark and photo-excited *R*_*xy*_ (left ordinate) and *R*_*xx*_ (right ordinate) vs. the magnetic field *B* to *B* = 2 Tesla. Here, the supplemental dc-current bias, *I*_*dc*_ = 0, and the frequency of microwave excitation for the photo-excited trace is *f* = 58 *GHz*. Note that the dark *R*_*xy*_ has been offset with respect to the photo excited *R*_*xy*_ trace for the sake of clarity. The Fig. 1(a) shows that the dark and photo-excited traces are nearly the same. Indeeed, subtracting the photo-excited data from the dark data for both *R*_*xy*_ and *R*_*xx*_ shows a vanishing residual. These residuals shown as ${\rm{\Delta }}{R}_{xy}={R}_{xy}^{(photo-excited)}-{R}_{xy}^{(dark)}$ and ${\rm{\Delta }}{R}_{xx}={R}_{xx}^{(photo-excited)}-{R}_{xx}^{(dark)}$ in Fig. 1(b) are vanishingly small in comparison to the as-collected signals. Thus, at first sight it looks like there is hardly a difference between the photo-excited and dark curves in the absence of a *dc*-current bias, although a close examination shows small Shubnikov-de Haas (SdH) in the residue since the SdH oscillations are slightly suppressed by the microwaves. The characteristic field for cyclotron resonance is labeled as *B*_*f*_ = 2πfm^*^/*e*, where *f* is microwave frequency, m^*^ is effective mass, *e* is the electron charge, and it is indicated by the dotted vertical line. Figure 1(c,d) show the transport results when a supplemental *dc*-current bias is applied to the sample. Here, when *I*_*dc*_ = 30 *μA*, the photo-excited *R*_*xy*_ shows evidence for Hall oscillations with plateau like features in comparison to dark *R*_*xy*_ for *B* ≥ 0.5 Tesla, see Fig. 1(c). Again, the dark *R*_*xy*_ has been offset with respect to the photo-excited *R*_*xy*_ for the sake of clarity. Concurrently, the *R*_*xx*_ trace shows strong *B*-periodic oscillations on top of the SdH oscillations, which were evident in *R*_*xx*_ of Fig. 1(a). [Such behavior is also observable in Fig. 2(a,d), which also suggest some harmonic distortion in the SdH oscillations under these experimental conditions]. The background subtracted Δ*R*_*xy*_ and Δ*R*_*xx*_ extracted from Fig. 1(c) have been plotted in Fig. 1(d). This figure demonstrates strong B-periodic oscillations in both *R*_*xy*_ and *R*_*xx*_ induced by the application of the *I*_*dc*_ in the presence of microwave photo-excitation for *B* > 0.25 Tesla. The maxima (minima) of Δ*R*_*xy*_ oscillations align with the minima (maxima) of Δ*R*_*xx*_ oscillations. An observable beat in the *B*-periodic oscillations, which did not show an obvious dependence on *f*, occurs for *B* ≈ 1.3 Tesla. This feature suggests the possibility of interference between two harmonic terms closely spaced frequency, and differing in frequency by ≤ 10%. Note that Δ*R*_*xy*_ and Δ*R*_*xx*_ oscillations are observed for *B* > *B*_*f*_.

Similar results are shown in Fig. 2 at other *f*. Figure 2(a,c) exhibit photo-excited and dark *R*_*xy*_ and *R*_*xx*_ curves at *I*_*dc*_ = 30 *μA* and *f* = 31 and 46 *GHz* respectively. As in Fig. 1(c), additional small B-periodic oscillations become evident on the *R*_*xy*_ and *R*_*xx*_ under the combined application of both the current bias and the microwave photo-excitation. The additional B-periodic features on the photo-excited *R*_*xy*_ in Fig. 2(a,c) have a distinct plateau-like appearance to them. Figure 2(b,d) show Δ*R*_*xy*_ and Δ*R*_*xx*_ at *f* = 31 and 46 *GHz* respectively. In both Fig. 2(b,d), B-periodic oscillations appear in Δ*R*_*xy*_ and Δ*R*_*xx*_ above *B*_*f*_. Note that the period of the these microwave- and current-bias-induced oscillations decreases with increasing microwave frequency.

**Figure 3:**
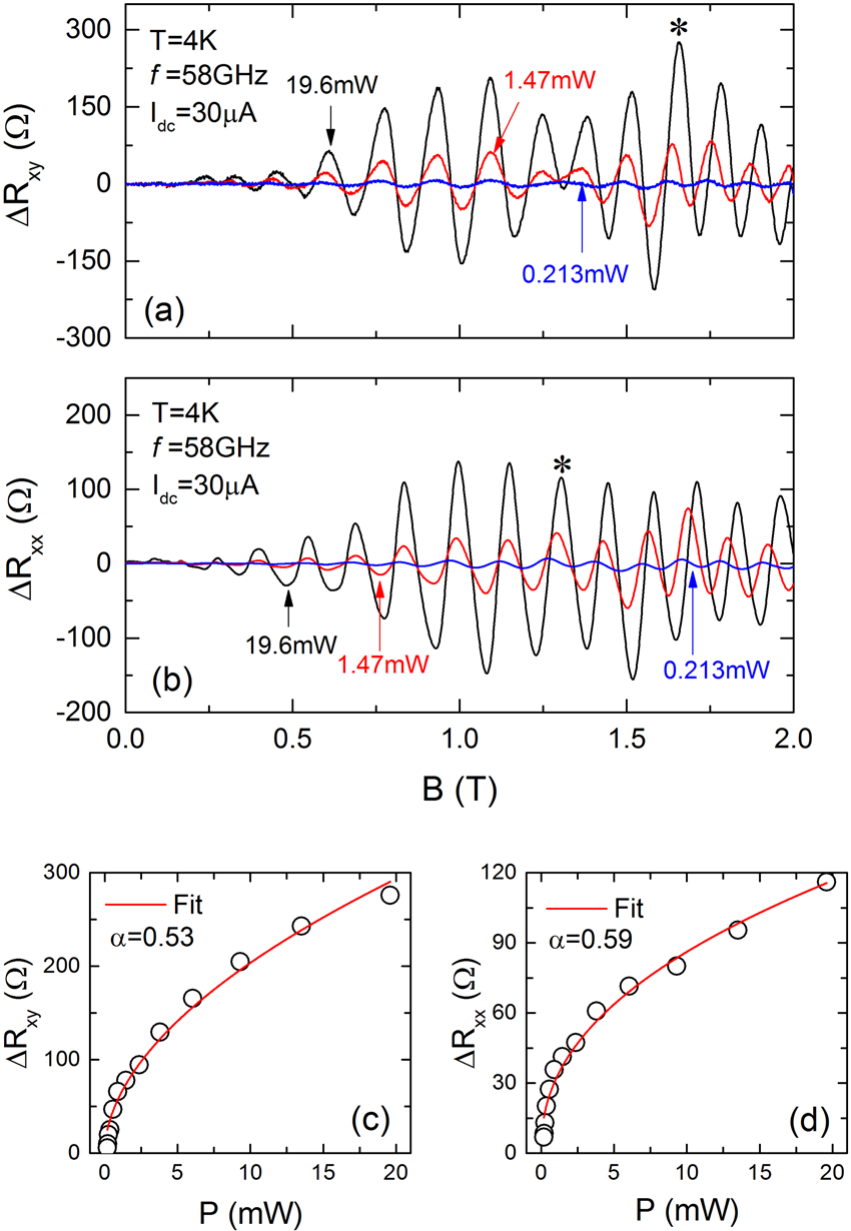
The microwave source-power *P* dependence data for (**a**) Δ*R*_*xy*_ and (**b**) Δ*R*_*xx*_ vs. *B* at *I*_*dc*_ = 30 *μA* and *f* = 58 *GHz* suggests that the amplitude of oscillations increases with increasing *P*. At the same time, there is a slight shift in the *B* positions of oscillatory extrema with increasing *P*. The amplitudes of oscillatory maxima (labeled with an asterisk) of (**c**) Δ*R*_*xy*_ and (**d**) Δ*R*_*xx*_ as a function of *P* suggests a non-linear increase in the amplitude with *P*. The function, Δ*R* ∝ *P*^*α*^, serves to fit the experimental data. The extracted *α*, indicated in Fig. 3(c,d) confirm a sub-linear relationship between amplitude and *P* for both Δ*R*_*xy*_ and Δ*R*_*xx*_ oscillations.

Figure 3 examines the microwave source power, *P*, dependence of Δ*R*_*xy*_, in panel (a), and Δ*R*_*xx*_, in panel (b), vs. *B* at *f* = 58 *GHz*. From Fig. 3(a,b), it is apparent that both Δ*R*_*xy*_ and Δ*R*_*xx*_ oscillation amplitudes are enhanced by increasing *P*. A closer investigation suggests that the *B* positions of oscillatory extrema move toward high *B* as *P* increases. Panel (c) and (d) exhibit the amplitudes of specified oscillatory maximum (labeled with an asterisk) of Δ*R*_*xy*_ and Δ*R*_*xx*_ as a function of *P* at *f* = 58 *GHz*. The data illustrate a sub-linear relation between the amplitude and *P*. A power law function, Δ*R* ∝ *P*^*α*^, has been applied to the experimental data to extract *α* characterizing the increase in the amplitude with *P*. The preliminary results indicate that *α* ≈ 0.55 ± 0.1, which suggests that the oscillation amplitude could be sensitive to the magnitude of the microwave electric field, *E*, since *E*∝*P*^0.5^ ^19^.

**Figure 4:**
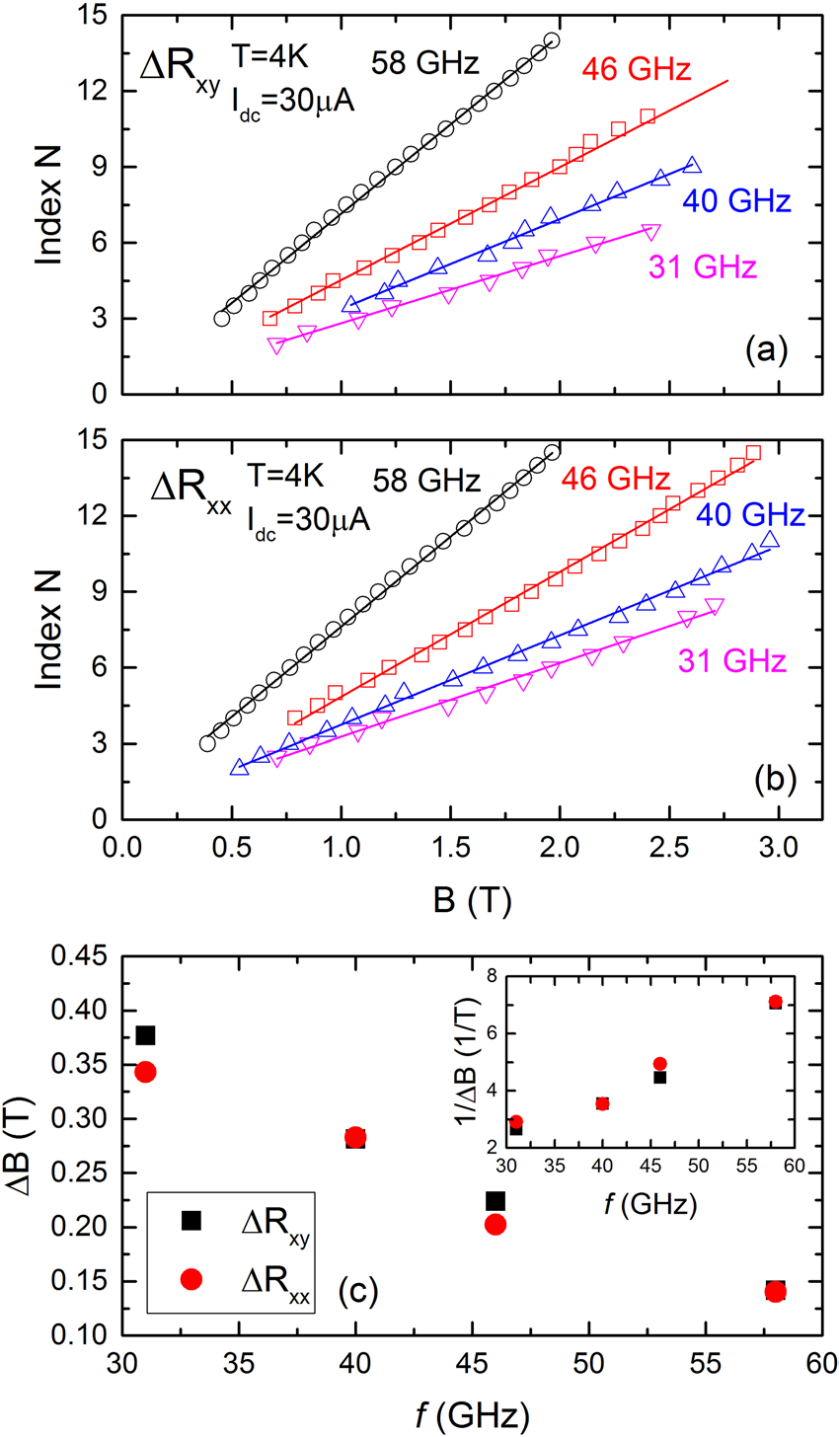
This figure shows plots of the extremal index vs. *B*, for both the Δ*R*_*xy*_ (panel (a)) and the Δ*R*_*xx*_ (panel (b)). Here, oscillatory maxima have been assigned with integer values while minima have been assigned half integer values. These half-cycle plots have been shown for *f* = 31, 40, 46, and 58 *GHz*. Note the straight line fits through the data. The results confirm *B*-periodicity in the *I*_*dc*_ induced magneto-oscillations. (**c**) This panel shows plots of Δ*B* vs. *f* and, in the inset, 1/Δ*B* vs. *f* extracted from Fig. 4(a,b).

To determine the periodicity of the *B*-periodic oscillations, the oscillatory maxima of *R*_*xy*_ and *R*_*xx*_ were assigned to integer values and the oscillatory minima to half-integer values. The plots of the oscillation index, *N* as a function of the extremal *B*-value for the oscillatory *R*_*xy*_ and *R*_*xx*_ for *f* = 31, 40, 46, and 58 *GHz* are exhibited in Fig. 4(a,b); these plots confirm a linear relationship indicating that the *R*_*xy*_ and *R*_*xx*_ oscillations are periodic-in-*B*. The period, Δ*B*, of the *R*_*xy*_ and *R*_*xx*_ oscillations as a function of *f* are plotted in Fig. 4(c), while the inset shows a plot of 1/Δ*B* vs. *f*. Since data points are shown at only four frequencies in Fig. 4(c), it is difficult to clarify the functional dependence of the oscillatory effect on the microwave frequency from these measurements. Studies at higher frequencies appear necessary to further investigate the relationship between the period of oscillations and microwave frequency.

## Discussion

Plasmons are collective excitations of electronic system that arise upon displacing electrons from their equilibrium positions with respect to the background positive charge^54^. A GaAs/AlGaAs 2DES is expected to exhibit a collective plasmon response in the absence of a magnetic field, i.e., *B* = 0, following the dispersion ${\omega }_{p}^{2}=n{e}^{2}k\mathrm{/2}{\varepsilon }_{eff}{\varepsilon }_{0}{m}^{\ast }$, where *ω*_*p*_ is the plasmon frequency, *n* is the electron density, *e* is the electron charge, *k* is the plasmon wave vector, m^*^ is the effective mass, and for the GaAs/AlGaAs 2DES, *ε*_*eff*_ = (*ε*_*GaAs*_ + *ε*_*vac*_)/2, with *ε*_*GaAs*_ = 12.8, and *ε*_*vac*_ = 1^55^. The application of a transverse magnetic field leads to a hybridization of cyclotron resonance with this plasmon, producing the (bulk) magnetoplasmon which follows ${\omega }_{mp}^{2}={\omega }_{p}^{2}+{\omega }_{C}^{2}$^54^. In a strip or Hall bar geometry, the length scale established by the boundary helps to determine the quantization condition or allowed values for the plasmon wavevector, *k*. Vasiliadou *et al*. investigated the transport signature of this phenomenon in Hall bars and found that their data could be described by *k* = *π*/*W*, where *W* is the width of the device^55^. This suggests that the finite sized specimen should exhibit the magnetoplasmon or plasmon shifted cyclotron resonance (*hf* = *ħω*_*mp*_) in place of the bare cyclotron resonance (*hf* = *ħω*_*C*_). In addition to the lowest mode, it is possible to also have additional allowed plasmon modes at wave vectors *k*, i.e., *k* = *nπ*/*W*, with *n* = 2, 3, 4…. Then, one expects additional magnetoplasmon branches to also leave behind a signature in transport. However, all these magnetoplasmon resonances would be expected to occur at magnetic fields below the bare cyclotron resonance magnetic field at a fixed frequency, *f*, for photoexcitation, i.e., *B* ≤ *B*_*f*_ = 2πfm^*^/*e*.

In addition to bulk plasmons, there exist edge plasmons that occur in bounded specimens. In contrast to the bulk plasmons, the mode frequencies of edge magnetoplasmons decrease with increasing magnetic field and follow the relation ${\omega }_{emp}={\mathrm{((2}}^{\mathrm{1/2}}\mathrm{/3)(3}{\omega }_{p}^{2}+{\omega }_{C}^{2}{)}^{\mathrm{1/2}}-{\omega }_{c}) \sim {\omega }_{p}^{2}/{\omega }_{c}$^54^. As with bulk magnetoplasmons, many edge magnetoplasmon modes are possible, one for each allowed value for *k* in the bounded specimen

As mentioned, our study reveals strong B-periodic oscillations in the Hall resistance that go together with the *R*_*xx*_ oscillations. Remarkably, the features in the *R*_*xy*_ trace even have a plateau like appearance associated with them, see Fig. 2(a,c). Such B-periodic oscillations in the Hall resistance have not been reported before, to our knowledge. On the other hand, the *B*- periodic oscillations in *R*_*xx*_ appearing in this study under microwave excitations are similar to the *B*-periodic oscillations in *R*_*xx*_ discussed in ref.^49^. Further, in our study, it appears vitally important to apply a supplementary current, i.e., a dc-current bias, to realize the *B*-periodic oscillations. It is the moderate microwave excitation in the presence of the dc-current bias that helps to bring out the *B*-periodic oscillations in both *R*_*xy*_ and *R*_*xx*_. Although such data from our study have not been shown here, the period of observed *B*-periodic oscillations in the *R*_*xx*_ did not depend on the spacing of the voltage contacts, as in the work of Stone *et al*.^52^. Early work claimed an edge magnetoplasmon origin for such *B*-periodic *R*_*xx*_ oscillations based on the dependence of the period on microwave frequency, electron density, and distance between potential contacts. As mentioned, such oscillations were attributed to the interference of coherently excited edge magnetoplasmons (EMP) at adjacent diagonal voltage contacts along the periphery of the sample^49,50,53^. Yet, the independence of the period on the potential probe distance^52^ seems to be, at first sight, in variance with expectations based on the edge magnetoplasmon model. ref.^52^ suggested, however, that, given the long decay length of edge magnetoplasmon modes, such can propagate along the whole edge around the sample as a consequence of the high- sample mobility. We note that even in the high mobility sample, thermal dissipation at the source and drain contacts may not support the propagation of edge magnetoplasmons across current contacts. That is, the EMP’s on opposite edges of the sample, on either side of the line connecting the source and the drain, are most likely decoupled. In this situation, it is difficult to understand the observed similarity between the magnetooscillations in the *R*_*xx*_ and *R*_*xy*_ in our measurements since the *R*_*xy*_ contacts lie on opposite edges while the *R*_*xx*_ contacts lie on the same edge. The requirement of a *dc*-bias for the observability of the effect, together with the improved observability of the effect at higher bath temperatures, *T* ≈ 4 K, suggests that the *dc*-bias serves to heat the electron system, and the current heating helps to bring about the obervability of the effect. Certainly, the observed effects are fascinating and further measurements are being carried out to understand their origin, and the role of the *dc*-bias in the electronic system^30,31^.

In summary, we have observed a *dc* current bias induced *B* - periodic Hall-oscillations that go together with longitudinal magnetoresistance oscillations in the GaAs/AlGaAs 2D electron systems under combined microwave- and *dc* bias- excitation. As noted, these *B*-periodic oscillations in *R*_*xy*_ go together with remarkable plateau-like features in the Hall resistance trace. The Hall and longitudinal magnetoresistance oscillations reveal similar period at given microwave frequency as their amplitude increases sub-linearly with the microwave power. The dependence of the observed effect to the *dc*– bias current offers a new method to study the *B*-periodic magnetooscillations with an easily controlled experimental parameter in a given specimen.

## Methods

### Sample Preparation

GaAs/AlGaAs heterojunctions were grown by molecular beam epitaxy and 200-*μm*-wide Hall bars were fabricated by optical lithography, and they included alloyed gold-germanium contacts. The specimen’s carrier density and mobility were *n*_*e*_ ≈ 2.4 × 10^11^ *cm*^−2^ and *μ* ≈ 11.6 × 10^6^ *cm*^2^⋅*V*^−1^⋅*s*^−1^ at 1.5 *K* respectively.

## Measurement Configuration

A Hall bar was mounted at the end of a 0.5′′-diameter stainless steel waveguide sample holder. The sample holder was immersed into pumped liquid helium. The temperature of the sample was controlled over the span 1.5 ≤ *T* ≤ 4 *K* by controlling the helium vapor pressure. The magnetic field, produced by a superconducting solenoid, was aligned along waveguide axis and perpendicularly to the sample. Microwaves were generated by a synthesizer over the frequency range 30 ≤ *f* ≤ 50 *GHz* at a source power 0.1 ≤ *P* ≤ 10 *mW* and a millimeter wave IMPATT diode source at *f* = 58 *GHz* with a maximal source power 20 mW. The *TE*_10_ mode microwaves excited by a probe-coupled antenna launcher was transported through the waveguide onto the sample and the electric field was oriented along the Hall bar long axis. The Hall voltage, *V*_*xy*_, and diagonal voltage, *V*_*xx*_, were collected using a four-terminal lock-in technique with an low-frequency *ac* current, *I*_*ac*_, flowing along the Hall bar; as indicated in Fig. 1 inset. A supplemental *dc* current, *I*_*dc*_, was applied along with *I*_*ac*_ for a portion of the measurements.
